# Structural dynamics and pH‐regulated membrane binding of the TRAF2‐NC trimer

**DOI:** 10.1002/pro.70817

**Published:** 2026-10-06

**Authors:** Federica Cossu, Serena Virili, Bianca Braggiotti, Francesco Bonì, Federico Ballabio, Toni Giorgino, Mario Milani, Giampiero Mei, Almerinda Di Venere, Eloise Mastrangelo

**Affiliations:** ^1^ Institute of Biophysics, National Research Council (IBF‐CNR) Milan Italy; ^2^ Department of Experimental Medicine Tor Vergata University of Rome Rome Italy; ^3^ Institute of Structural Biology, Molecular Targets and Therapeutics Center Helmholtz Munich ‐ Deutsches Forschungszentrum Für Gesundheit Und Umwelt (GmbH) Neuherberg Germany

**Keywords:** lipid interaction, membrane binding, structural dynamics, TNF receptor signaling, TRAF2, trimerization

## Abstract

Tumor necrosis factor receptor‐associated factor 2 (TRAF2) is a key adaptor in TNF receptor‐signaling that operates as a trimeric assembly. Here we investigate the structural and functional properties of a TRAF2 construct spanning the trimerization and receptor‐binding domains (TRAF2‐NC, residues 266–501). According to SAXS data, under physiological conditions, TRAF2‐NC preserves a stable trimeric structure with signs of conformational flexibility. Conversely, in a mildly acidic environment (pH 5.7), the protein adopts a less compact and more heterogeneous conformation. In this condition, fluorescence energy transfer measurements reveal an increased membrane‐binding propensity in the presence of ganglioside GM1. Together, these results indicate that TRAF2 oligomerization is modulated by pH‐dependent conformational changes, suggesting that increased structural flexibility and partial exposure of hydrophobic regions facilitate membrane association under stress conditions, and providing a mechanistic link between structural dynamics and TRAF2 function.

## 1 INTRODUCTION

Tumor necrosis factor receptor‐associated factor 2 (TRAF2) is a central adaptor protein in tumor necrosis factor receptor (TNFR) signaling, orchestrating inflammatory pathways, immune responses, and programmed cell death (Borghi et al. 2016; Rothe et al. 1994). TRAF2 mediates signaling downstream of integral membrane TNFRs that oligomerize upon cytokine binding, triggering intracellular signaling cascades involved in inflammation and apoptosis (Bremer 2013; Chen and Goeddel 2002). Acting as a molecular bridge, TRAF2 links receptor complexes to downstream effectors such as NF‐κB and MAPK pathways, regulating diverse physiological and pathological responses, from immunity to cancer (Albini et al. 2025; Cabal‐Hierro et al. 2014; Liang et al. 2023; Siegmund et al. 2022; Zhu et al. 2023).

TRAF2 is composed of an N‐terminal RING domain (residues 1–83), five zinc fingers (84–266), a coiled‐coil domain (TRAF2‐N, 267–328), and a globular C‐terminal domain (TRAF2‐C, 329–501) (McWhirter et al. 1999; Park et al. 1999). The TRAF2‐N domain mediates trimerization, which is essential for high‐avidity binding to trimeric TNFRs and subsequent signal transduction. The TRAF2‐C domain engages signaling partners and adopts a β‐sandwich fold characteristic of the TRAF protein family. Crystal structures of the C‐terminal region revealed a mushroom‐shaped trimeric architecture, observed both in the unbound form and in complexes with receptor motifs such as CD40 and CD137 (Jang et al. 1998; Zapata et al. 2018).

Consistent with its role as a signaling hub, TRAF2 participates in numerous crucial interactions, engaging with multiple partners, including TNFRs (Ye et al. 1999), TRADD (Park et al. 2000), TRAF1 (Zheng et al. 2010), A20 (Li et al. 2009), RIP1 (Webster and Vucic 2020), glutathione transferase P1‐1 (De Luca et al. 2014), and cellular IAPs (cIAPs) (Zheng et al. 2010). Within the TNF receptor signaling network, these interactions contribute to the assembly of higher‐order signaling complexes that regulate downstream NF‐κB activation (Vince et al. 2009; Walczak 2013). The interaction with cIAPs is particularly relevant and represents a promising pharmacological target for anticancer therapy (Cossu et al. 2020; Cossu et al. 2021). Importantly, many of these interactions are influenced by the oligomeric state of the protein: TRAF2 forms homotrimers but can also assemble into TRAF1:(TRAF2)_2_ heterotrimers with enhanced affinity for cIAPs. Conversely, dissociation of the TRAF2‐C domain can lead to monomeric or dimeric forms that can interact with membranes (Minicozzi et al. 2022). Using fluorescence energy transfer studies, Di Venere et al. showed that the TRAF2‐C domain associates with lipid bilayers through specific interactions (Di Venere et al. 2018). In particular, the presence of the ganglioside GM1 significantly decreases the protein‐membrane dissociation constant and produces vesiculation of POPC unilamellar vesicles (Ceccarelli et al. 2017; Di Venere et al. 2018). These observations support the idea that lipid components of the membrane may contribute to the subcellular regulation of TRAF2 within cholesterol‐rich microdomains, which are known to function as dynamic signaling platforms for receptors and adaptor proteins (Pike 2003; Simons and Toomre 2000). More recently, it has been shown that TRAF2‐C binds GM1 both in vitro and in human cells under neutral and mildly acidic conditions, such as those encountered during apoptosis (De Luca et al. 2024). Under acidic pH, the TRAF2‐C predominantly adopts a monomeric state and induces deformation of GM1‐containing vesicles, suggesting that pH‐dependent structural transitions may regulate membrane association during cell death.

Despite these advances, several aspects of TRAF2 structural regulation remain unclear. In particular, the structure of the full‐length protein has not yet been determined, limiting our understanding of how the different domains cooperate in activation, membrane binding, and signal integration. Moreover, the conformational dynamics of the trimeric assembly are still poorly characterized. Computational analyses have suggested that the TRAF2‐C trimer may display asymmetric behavior, in which two subunits per time act as a single cluster, with a higher number of interface connections compared to the third one (Minicozzi et al. 2021). The comparison with the simulations of a longer TRAF2‐C construct (+23 amino acids) has shown that the protein tail might influence the globular, C‐terminal domains, suggesting that the coiled‐coil interactions can also play an important regulatory function (Erba et al. 2023). To investigate how the stability and membrane‐binding capability of TRAF2 depend on the length of the N‐terminal region, we produced a construct spanning residues 266–501 (TRAF2‐NC), encompassing both the trimerization (TRAF2‐N) and receptor‐binding (TRAF2‐C) domains. Using a combination of structural, biophysical, and biochemical approaches, we examined its oligomeric organization in solution, its conformational behavior under different environmental conditions, and its interaction with lipid membranes. These experiments provide insight into how conformational plasticity and oligomerization may contribute to the regulation of TRAF2 function.

## 2 RESULTS

### 2.1 TRAF2‐NC production, stability, and preliminary structural characterization

The TRAF2‐NC construct (residues 266–501) was successfully cloned into a pET21(A) vector and expressed in *E. coli* C41(DE3) cells. The protein was obtained at high purity, as confirmed by SDS‐PAGE (Figure S1, Supporting Information).

To assess the suitability of the purified protein for downstream biophysical characterization and to identify optimal experimental conditions, its thermal stability was evaluated under different buffer compositions and pH values (Table S1). The data obtained demonstrate that the protein remains quite stable in different conditions (melting temperatures in the range 51–56°C) except for sodium citrate buffer at pH 4.

To characterize the oligomeric state of TRAF2‐NC in solution and to assess sample homogeneity, Flow‐Induced Dispersion Analysis (FIDA) was performed as described (see section 4). The results of two different runs are shown in Figure 1a. The presence of a single, reproducible elution profile demonstrates that a monodisperse oligomeric species was present in solution, whose estimated hydrodynamic diameter was *D* = 8.83 ± 0.06 nm.

**FIGURE 1:**
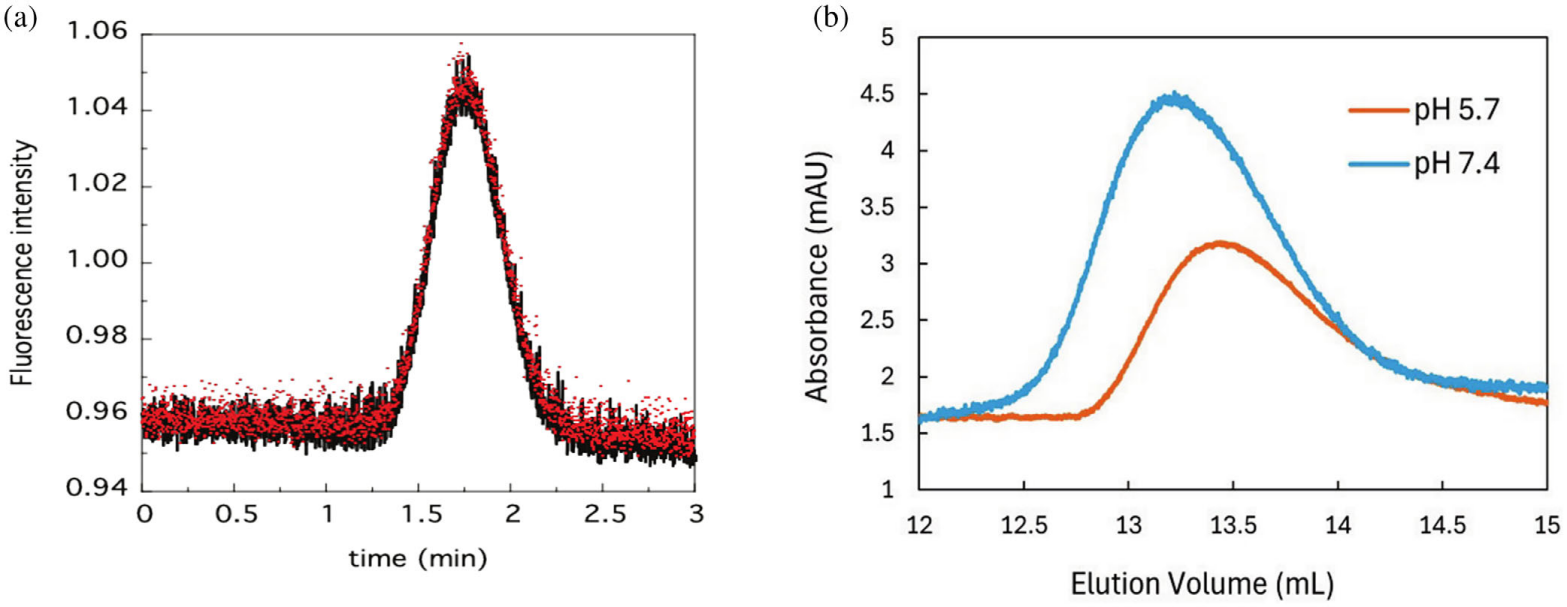
TRAF2‐NC oligomeric analysis. (a) FIDA elution profile of TRAF2‐NC ([M] = 11 μM) of two independent runs; (b) SEC analysis: zoom peak of TRAF2 elution volume (Ve) at pH 7.4 (blue line) and pH 5.7 (orange line).

An independent evaluation of the protein oligomerization state was obtained through Size‐exclusion chromatography (SEC) at a protein concentration of 10 μM and neutral pH. The analysis revealed that TRAF2‐NC predominantly elutes as a single oligomer (Figure 1b). The average elution volume (Ve) corresponds to a calculated MW = 102.6 kDa (from the standard calibration curve; see section 4) consistent with an elongated trimer (theoretical MW = 85.8 kDa). A comparable elution profile was observed at pH 5.7 (Ve = 13.44; calculated MW = 94.7).

The protein size was also estimated by light scattering analysis (Figure 2). In this case, the fit of the particle distribution profile at pH 7.4 yielded an average diameter value of *D* = 9.1 ± 0.2 nm (5 μM), in good agreement with the FIDA results.

**FIGURE 2:**
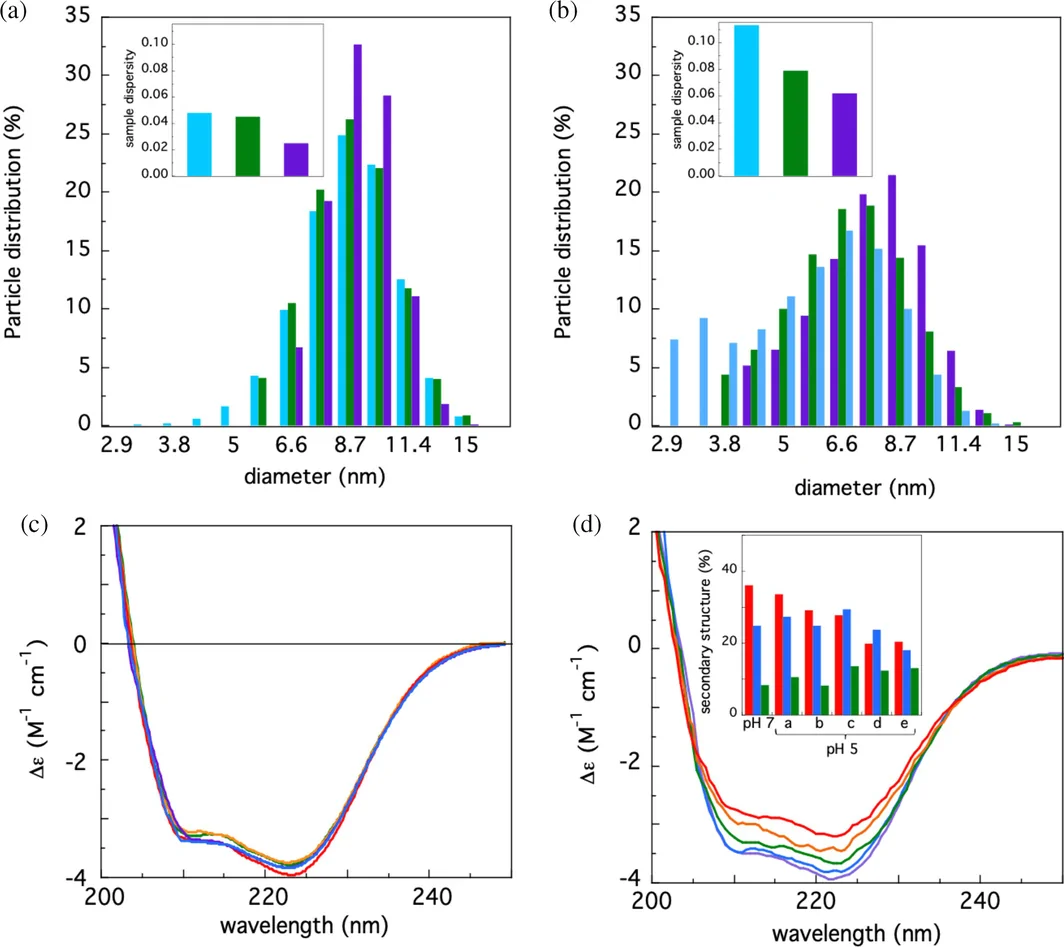
Biophysical characterization of TRAF2‐NC. (a, b) Light scattering of TRAF2‐NC at pH 7.4 (a) and pH 5.7 (b) at different monomer concentrations, namely [M] = 5 (purple bars), 2 (green), and 0.5 μM (cyan). The corresponding sample dispersity values are reported in the two insets. (c, d) Circular dichroism spectra of TRAF2‐NC at pH 7.4 (c) and pH 5.7 (d) at different concentrations ([M] = 16 (purple line), 8 (blue), 4 (green), 2 (orange), and 1 μM (red)). The percentage number of helices, sheets and turns (red, blue and green bars, respectively) are shown in the inset, in the case of pH 7.4 and pH 5.7 (a = 16 μM; b = 8 μM; c = 4 μM; d = 2 μM; e = 1 μM).

To test the protein dissociation propensity, the measurements were repeated at lower TRAF2‐NC concentration (2 and 0.5 μM). The data show a slight shift and a modest widening of the particle distribution (Figure 2a), indicating that TRAF2‐NC is mainly trimeric at neutral pH. In contrast, TRAF2‐C construct showed protein monomerization at mildly acidic pH (De Luca et al. 2024). To test whether a similar behavior characterizes TRAF2‐NC, light scattering measurements were repeated at pH 5.7. Indeed, the broader distribution obtained (Figure 2b) indicates the presence of smaller molecular species (i.e., dimers and monomers), whose amount increases at low concentration, as expected if subunits dissociation occurs.

Overall, these data show that TRAF2‐NC forms a stable homotrimer at neutral pH, while mildly acidic conditions increase structural heterogeneity and promote partial destabilization of the trimeric assembly.

To investigate the secondary structure of TRAF2‐NC and its dependence on oligomeric state, circular dichroism (CD) spectroscopy was performed under two complementary conditions: (i) as a function of protein concentration at different pH values, and (ii) as a function of temperature. First, far‐UV CD spectra were recorded at varying protein concentrations (1–16 μM) at pH 7.4 and pH 5.7 (Figure 2c,d). At neutral pH, the TRAF2‐NC spectra at different concentrations were superimposable (Figure 2c), indicating that in that range the protein maintains the same conformation. In contrast, at pH 5.7 (Figure 2d) a loss of the overall CD signal was obtained at lower protein concentration, suggesting that the secondary structures are influenced by the oligomerization state of the protein. The analysis of the secondary structure distribution (inset of Figure 2d) shows a reduction of α‐helical content by protein dilution, suggesting a partial loss of the coiled coil structure in the monomeric protein.

To further assess the overall stability at pH 7.4 and 5.7, thermal denaturation experiments were performed by monitoring the CD signal as a function of temperature (Figure 3). Both the denaturation experiments showed a cooperative non‐reversible denaturation profile with average melting temperatures (*T*_M_) of 54.9 ± 0.7°C and 51.3 ± 0.7°C, at pH 7.4 and 5.7, respectively. In both cases, the ellipticity remained constant up to 45°C, demonstrating that the protein secondary structure is stable at physiological temperature, although acidification promotes the destabilization of alpha‐helices.

**FIGURE 3:**
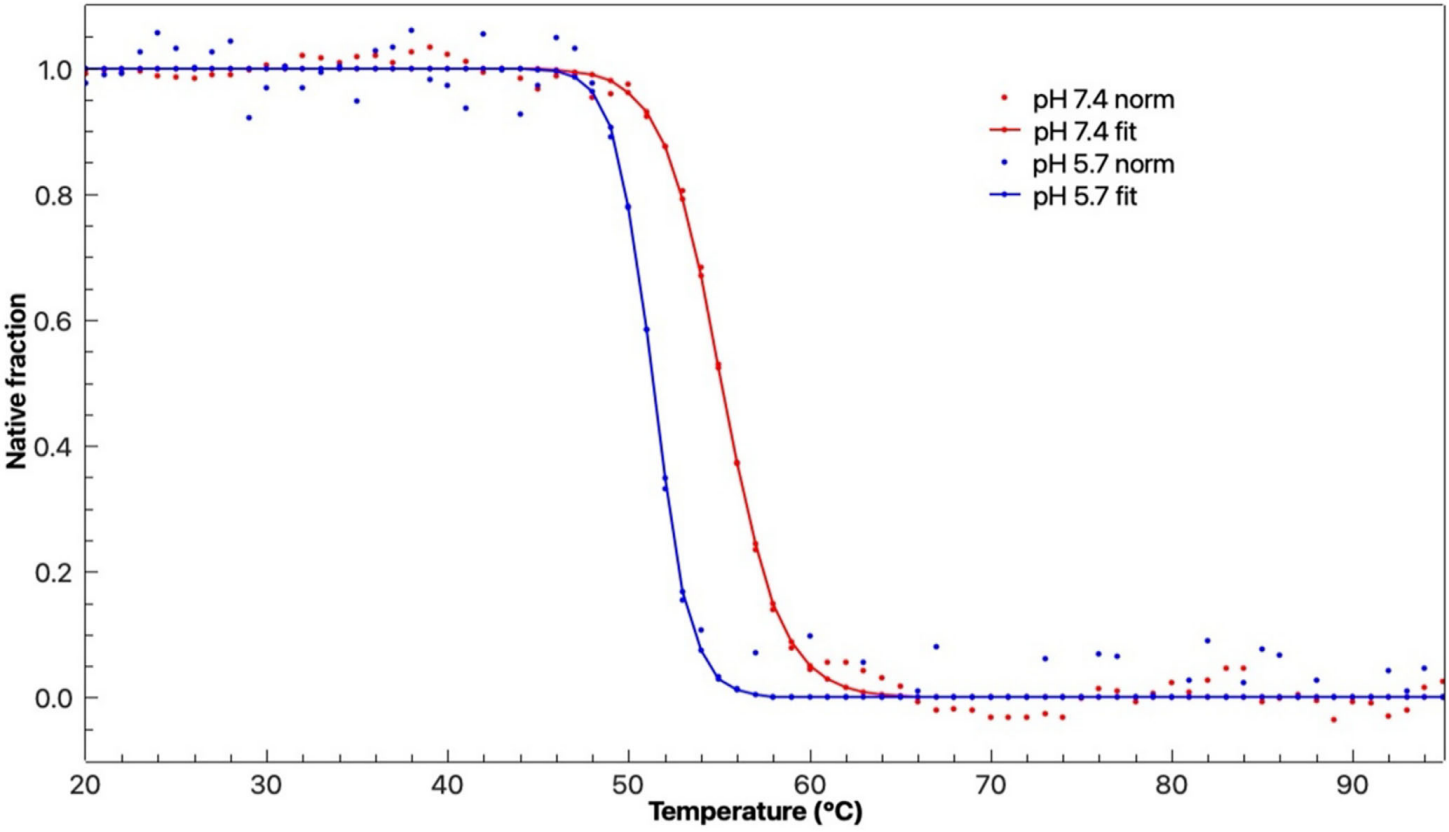
Normalized thermal denaturation curves of TRAF2‐NC. TRAF2‐NC at pH 7.4 denaturation profile in red; TRAF2‐NC at pH 5.7 denaturation profile in blue.

The protein tertiary structure and the conformational compactness have been characterized by intrinsic steady‐state fluorescence, upon excitation at *λ*_ex_ = 275 nm. In a folded protein, the contribution of tyrosine residues to the emission signal is generally negligible, due to an efficient energy transfer process, in which tryptophan(s) are involved as acceptors. Indeed, this is what was observed at pH 7.4 measuring the protein fluorescence as a function of temperature (from 10 to 55°C) (Figure 4a). The main features of the spectrum such as the width (FWHM) and the shape (F_322_/F_308_) (Figure 4a, inset) do not exhibit significant changes up to ≈40–45°C, in agreement with CD measures. On the contrary, the spectra recorded at pH 5.7 (Figure 4b) display a stronger dependence on temperature, with increased FWHM and a shoulder at 308 nm (Figure 4b, inset). This last feature, typical of tyrosine emission, indicates that the combined effect of pH and temperature leads to a less effective energy transfer, probably due to an increased Tyr‐Trp distance. To confirm such result, we recorded the fluorescence spectra at *λ*_ex_ = 292 nm, thus limiting excitation to tryptophan residues. The comparison of the normalized spectra at 10 and 45°C at the two different excitation wavelengths is shown in Figure 4c,d, respectively. The temperature dependent increase in fluorescent emission at shorter wavelengths demonstrates a loosening of the protein compactness at pH 5.7, in agreement with light scattering and CD measurements (Figure 2b,d).

**FIGURE 4:**
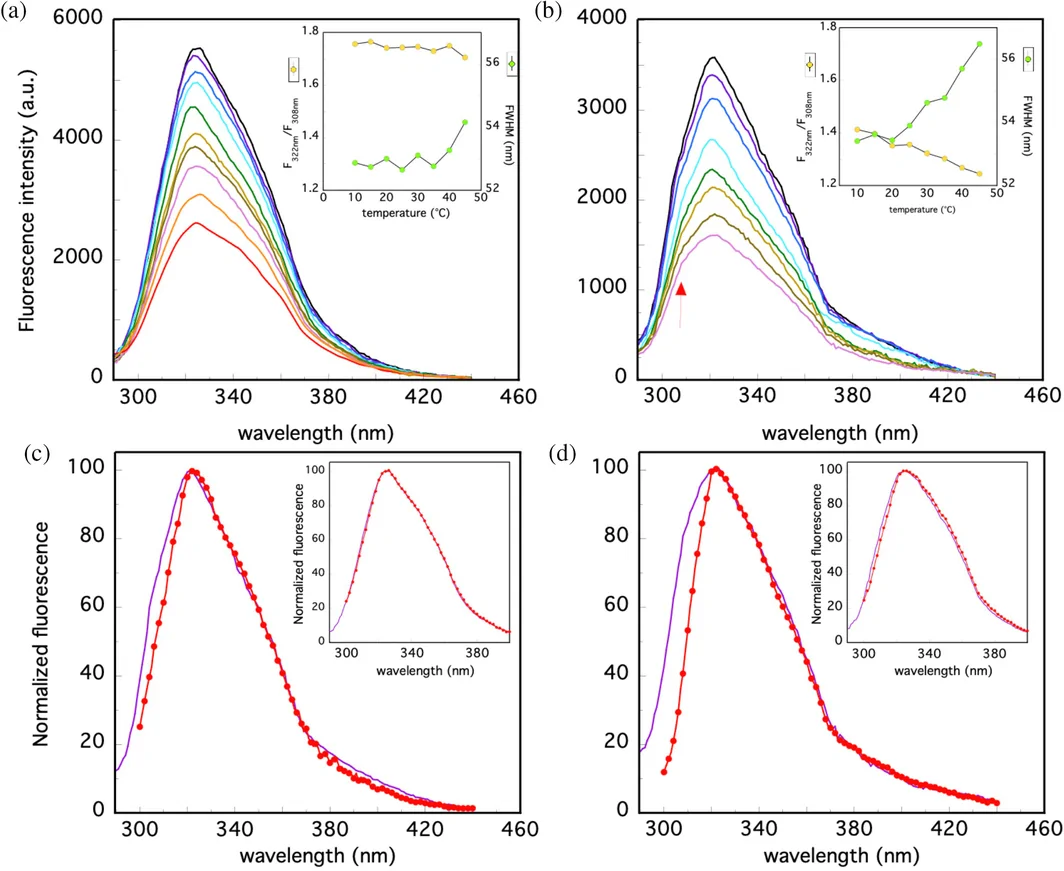
Temperature‐dependent fluorescence analysis of TRAF2‐NC. Fluorescence spectra of TRAF2‐NC (*λ*_ex_ = 275 nm) as a function of temperature, at pH 7.4 (a) and 5.7 (b). In this last case, a red arrow indicates the shoulder occurring at 308 nm (see text). The spectra were recorded every 5°C, in the range 10–55°C (from black to red). In the insets, the dependence of the protein spectral features (namely the Full Width at Half Maximum, FWHM in green, and the fluorescence ratio F_322_/F_308_, in yellow) are reported. The normalized spectra at pH 5.7 are shown in (c) (10°C) and panel D (45°C), upon excitation at *λ*_ex_ = 275 nm (purple line) and at *λ*_ex_ = 292 nm (red line and symbol). In the two insets, the respective spectra at pH 7.4 are reported, for comparison.

### 2.2 SAXS and SAXS‐restrained molecular dynamics simulation

To confirm that TRAF2‐NC adopts a trimeric architecture in solution, we performed small‐angle X‐ray scattering (SAXS) analyses. SAXS measurements of the protein in batch (17 μM) yielded an Rg of 4.24 ± 0.05 nm with an estimated Mw of 84.6 kDa using water as reference (program *primus*; Manalastas‐Cantos et al. 2021), consistent with a trimeric assembly (program *gnom*; Svergun 1992; Dmax = 13 nm). SEC‐SAXS experiment (concentration of the protein estimated by UV absorbance at SEC peak ~35 μM) gave consistent parameters (Rg = 4.28 ± 0.05 nm and Bayesian Mw = 78.5 kDa) further supporting the trimeric state in solution (*gnom* Dmax = 15.4 nm). Low‐resolution ab initio modeling with the program *DAMMIN* (Svergun 1999) yielded an envelope fully compatible with an elongated trimeric assembly (Figure 1a,b; *χ* ^2^ = 1.3). The high‐resolution trimeric model generated by AlphaFold (pTM = 0.85, ipTM = 0.84) showed limited agreement with SEC‐SAXS data (up to Smax = 0.3 Å^−1^, program *crysol χ* ^2^ = 36.3; Franke et al. 2017), which improved upon inclusion of the His‐tag sequence in the model (*χ* ^2^ = 17.8). Accounting for conformational flexibility of the high‐resolution model through normal mode analysis (program *SREFLEX*; Panjkovich and Svergun 2016) markedly improved the fit (*χ* ^2^ = 2.5; up to Smax = 0.5 Å^−1^; Figure 5c,d). Overall, SAXS data demonstrate that TRAF2‐NC forms a stable homotrimer in solution whose global architecture is consistent with the AlphaFold trimeric model, while retaining intrinsic conformational flexibility rather than behaving as a rigid symmetric assembly (as also confirmed by the Kratky plot; not shown). SEC‐SAXS experiment at acidic pH (pH 5.7) showed an increased radius of gyration (Rg = 4.50 ± 0.05 nm; *gnom* Dmax = 15.7 nm) consistent with a slight opening of the trimeric assembly, suggesting that low pH exerts a perturbing effect on the structure.

**FIGURE 5:**
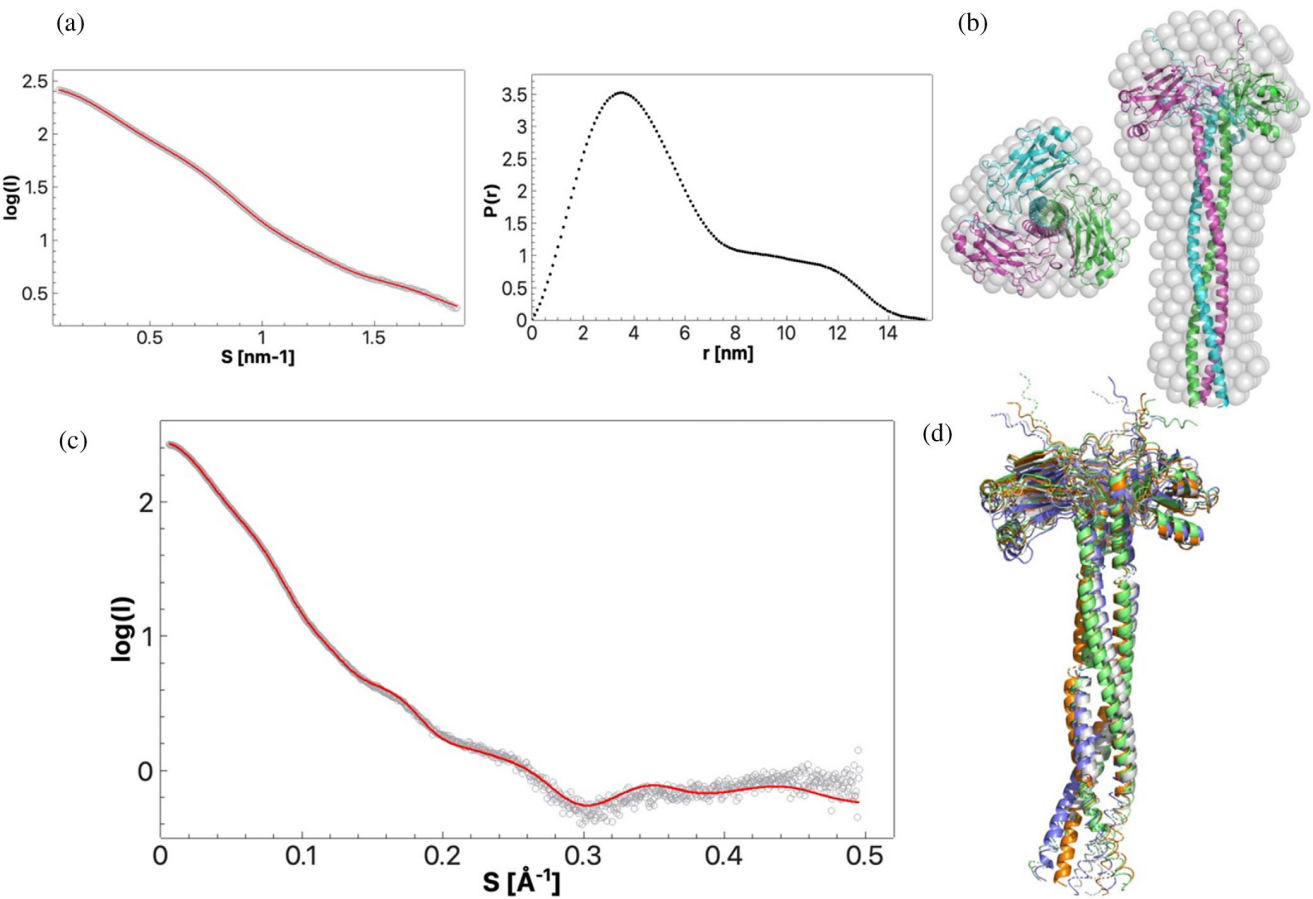
SEC‐SAXS analysis. (a) Low angle experimental SEC‐SAXS curve and P(r) calculated with program *Gnom*. (b) Low‐resolution model (transparent spheres) obtained with the program *Dammin*, superimposed to the high‐resolution AlphaFold model (cartoons). (c) Experimental SEC‐SAXS scattering curve fitted with the AlphaFold model after refinement by normal mode analysis using the program SREFLEX. (d) Cartoon representation of the SREFLEX‐refined model shown in (c). Protein models represented with program PyMOL (https://www.pymol.org/).

To further refine the structural model, SAXS‐restrained molecular dynamics (MD) simulations were performed starting from the AlphaFold3 trimer. Simulations were carried out using the CHARMM36m force field in GROMACS with PLUMED, and experimental SAXS data were applied as restraints over a 7‐ns SAXS‐guided structural refinement trajectory. SAXS profiles were calculated on‐the‐fly using the hybrid‐resolution hySAS approach, and only conformations exceeding a stringent correlation threshold (*R* > 0.999901) were retained. These conformations were independently evaluated with PEPSI‐SAXS over an extended *q* range. The representative refined model was selected on the basis of the best agreement with the experimental SAXS profile, rather than as the final frame of the trajectory, and yielded a *χ* ^2^ of 2.20. In summary, the refined model supports a trimeric assembly displaying subtle asymmetries and dynamic inter‐domain rearrangements, consistent with a flexible, solution‐state architecture. All SAXS‐compatible configurations retained by the selection procedure remained trimeric and adopted closely related arrangements, with a backbone RMSD of 2.4 ± 0.6 Å relative to the best‐scoring frame (2133 atoms; His‐tags excluded). Similarly, the three 300 ns unbiased control simulations showed a stable configuration, except for a limited “waving” of the coiled‐coil with respect to the C‐terminal trimeric head assembly (the TRAF2‐C domain). The trimer is held together by the coiled‐coil arrangement as well as the C‐terminal domain interfaces. Both the SAXS‐restrained and the unrestrained simulations showed a limited amount “untwisting” of the overall super‐helical bundle, from 270° (crystal and AlphaFold models) to 180° (MD) approximately; given the limited SAXS resolution and the consistent presentation between biased and unbiased simulations, this limited uncoiling is likely driven by the MD forcefield component, rather than SAXS biasing. The three unbiased MD yielded configurations with a minimum *χ* ^2^ 2.44, 3.37, and 4.79, respectively, with respect to the SAXS data (Figure S5).

### 2.3 TRAF2‐NC conformational flexibility and membrane binding

TRAF2 rotational correlation lifetimes are strongly connected to pH‐induced changes in the protein tertiary and quaternary structure, which, in turn, play a crucial role in bio‐membranes binding (De Luca et al. 2024). For such reason, we first monitored the TRAF2‐NC fluorescence dynamics at two different pH values (namely pH 7.4 and pH 5.7) and then tested, in the same condition, the protein interaction with synthetic liposomes. The results of phase‐shift and de‐modulation measurements are reported in Figure 6a,b for concentrated and diluted samples, respectively. The presence of two distributions of fluorescence lifetimes is a typical feature of TRAF2, and it arises from the heterogeneous environment of the aromatic amino acids contained in the protein C‐terminal domain (Ceccarelli et al. 2015).

**FIGURE 6:**
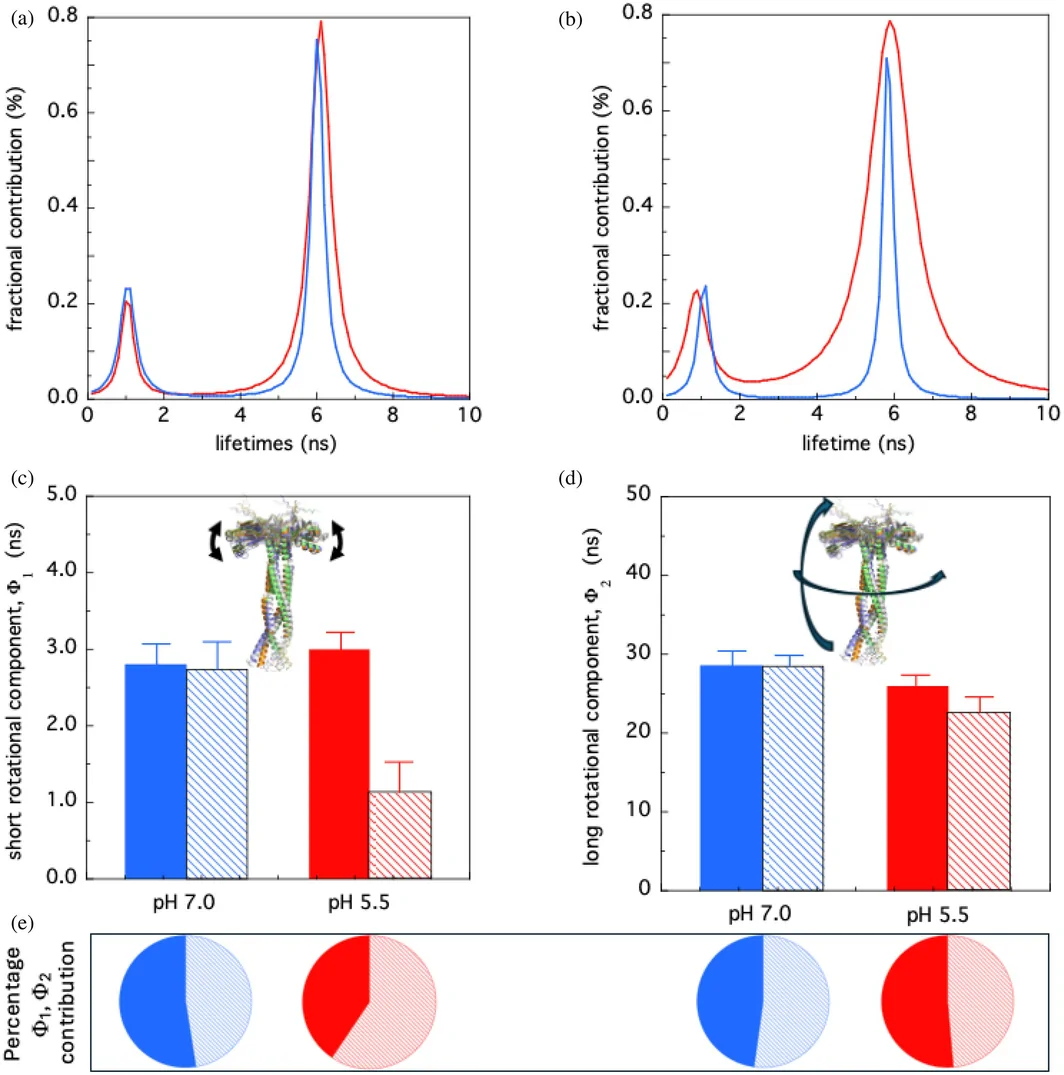
Fluorescence lifetime and rotational dynamics of TRAF2‐NC. Fluorescence lifetime distributions (panel a [TRAF2‐NC] = 12.0 μM and panel b [TRAF2‐NC] = 6.5 μM) of TRAF2‐NC at pH 7.4 (blue line) and 5.7 (red line). The results of dynamic fluorescence anisotropy measurements are reported in (c) (short rotational component) and (d) (long rotational component). Measurements have been performed both on the concentrated (filled bars) and diluted samples (dashed bars) used in lifetime experiments (a, b). In (e) the changes in the fractional contribution of each correlation time upon dilution is reported.

At high protein concentration, the measurements at two different pH yielded almost superimposable results, that is, two components (centered around ≈1 and ≈6 ns) with similar fractional contribution (20% and 80%, respectively). Interestingly, the FWHM of the longer component is slightly larger at pH 5.7 (Figure 6a), diagnostic of a more heterogeneous environment surrounding the aromatic residues. At low protein concentration the shape of the lifetime distributions is maintained at pH 7.4, while both lifetime components are significantly affected by dilution in an acidic buffer (Figure 6b). This result indicates that a larger number of conformational substates populates the protein tertiary structure at pH 5.7. The concentration dependence suggests a slight loosening of quaternary interactions consistent with the broader light scattering profile reported in Figure 2b. To further analyze both local and overall protein motions, we evaluated fluorescence anisotropy dynamics measuring the rotational correlation lifetimes through polarized light (Lakowicz 1999). As reported in Figure 6c,d two rotational lifetimes are required to fit the anisotropy fluorescence decay. The short component (Φ_1_ ≤ 3 ns) reflects fast movements of the C‐terminal domain's regions containing the aromatic residues (no Trp or Tyr are present in the coiled‐coil portion of the TRAF2‐NC). The combined effect of acid pH and dilution induces to major changes in the local mobility, as demonstrated by the decrease of the Φ_1_ value (Figure 6c) and the increased contribution to the anisotropy fluorescence signal (Figure 6e). The longer component (Φ_2_ ≈ 30 ns) accounts instead for the rotational motion of the whole protein (the revolution time of a spherical molecule with similar molecular weight is expected to be ≈35 ns) (Cantor and Schimmel 1980). In this case, lowering the pH and the protein concentration leads to a 25% decrease in the Φ_2_ value (Figure 6d), suggesting an enhanced mobility of the C‐terminal domain and/or a partial dissociation of the subunits.

The TRAF2‐NC membrane binding ability has been tested through fluorescence energy‐transfer measurements between tryptophan residues and the pyrene molecules contained in POPC synthetic vesicles. The percentual decrease of the intrinsic fluorescence signal (Δ*F*/Δ*F*_max_) is reported in Figure 7 as a function of lipid concentration. The data demonstrate that binding is pH‐dependent, being less efficient at pH 7.4 (Figure 7, blue symbols). To get a more quantitative result, a nonlinear fit of the data was performed assuming that the change in the protein intrinsic fluorescence is proportionally related to the fraction of bound protein, as already proposed by Tatulian and co‐workers in the case of Phospholipase A2 (Qin et al. 2004; cf., section 4). The main parameter obtained through such procedure is the dissociation constant, *K*_d_, whose value resulted to be almost twice larger at pH 7.4 (≈21 μM) with respect to the experiment performed at pH 5.7 (≈12 μM).

**FIGURE 7:**
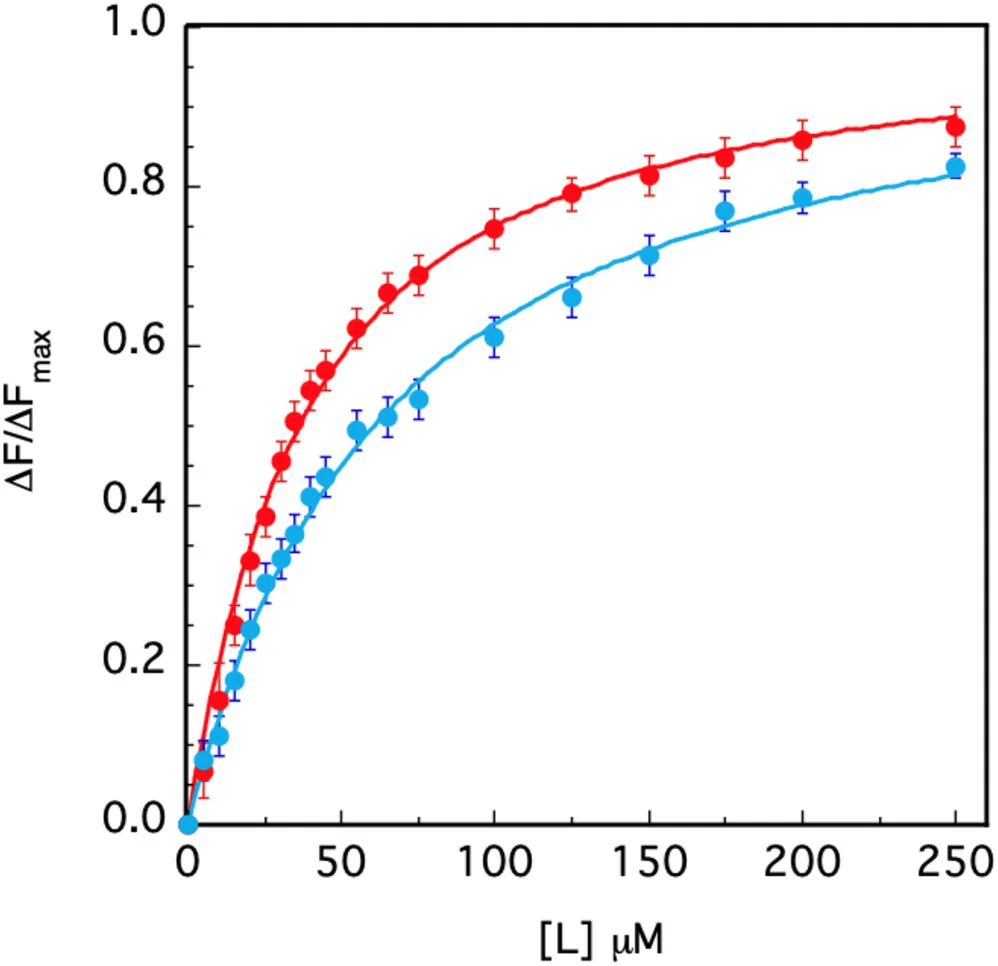
Binding curves of TRAF2‐NC to POPC containing LUVs at pH 7.4 (blue circles) and pH 5.7 (red circles). Data were obtained through FRET measurements between tryptophan residues and pyrene‐containing vesicles. The solid lines represent the best fit obtained as described in section 4 yielding *K*_d_ = 11.6 ± 0.4 μM and *K*_d_ = 21.1 ± 0.9 μM at pH 5.7 and pH 7.4, respectively.

## 3 DISCUSSION

The members of the TNF family act on cells through complex signal transduction mechanisms involving dozens of intracellular proteins with highly regulated functions. A common feature of this system is the ability of its components (i.e., TNFs, TNFRs, and TRAFs) to form trimeric oligomers. Indeed, single‐molecules studies have shown that TNFRs, which exist as monomers and dimers on the cell surface, aggregate upon interaction with trimeric TNF ligands (Karathanasis et al. 2020). On the cytosolic side, TRAF proteins are recruited to receptors and assemble into stable trimeric complexes through conserved domains (Park 2018, 2021). Overall, these findings indicate that TNF signaling relies on a dynamic monomer‐dimer‐trimer equilibrium involving ligands, receptors and transducers (Kucka et al. 2021; Zhao et al. 2020). In the case of TRAF2, such equilibrium has been previously investigated as function of temperature (Di Venere et al. 2021), pH (De Luca et al. 2024) and protein concentration (Erba et al. 2025), using short constructs corresponding to the C‐terminal domain. More recently, in silico simulations suggested that the TRAF2's tail contributes to protein dynamics and trimer stabilization (Erba et al. 2023). For this reason, here we analyzed a longer construct, which includes both the trimerization (TRAF2‐N) and the receptor‐binding (TRAF2‐C) domains, to better capture the structural behavior of the protein in solution. Our data show that TRAF2‐NC forms a stable homotrimer in solution at physiological pH. This is consistently supported by different techniques (SEC, FIDA, DLS, and SAXS), all pointing to a well‐defined oligomeric state. Additionally, SAXS and SAXS‐restrained MD simulations indicate that this trimer is not a rigid assembly but rather shows a certain degree of flexibility and some asymmetry between subunits. In this sense, our results extend what is known from crystal structures of the isolated TRAF2‐C domain, suggesting that the coiled‐coil region contributes to increased conformational dynamics in solution. When the pH is lowered to mildly acidic values, the overall trimeric organization is still largely maintained, but several experimental observations indicate that the system becomes more heterogeneous. In particular, at low pH the CD spectra become concentration‐dependent, suggesting a change in oligomeric state consistent with the broader structural distributions observed by SEC and DLS. Importantly, these results, together with the SAXS analysis showing a modest increase in the radius of gyration (ΔRg ~0.22 nm), indicate a weakening of inter‐subunit interactions without complete dissociation of the trimer. At low pH, the thermal destabilization of alpha helices observed with CD (Tm ~51°C) occurs before the complete unfolding of the protein (observed with thermofluorescence; Tm ~53°C), suggesting that the acidic condition negatively affects the coiled‐coil structure, possibly inducing the partial dissociation of the trimer.

Hints of the non‐optimal trimeric assembly of the coiled‐coil region at low pH are observed in the B chains of two crystal structures, that is, 3M0A and 3M06, around residue 303–309 and 307–312, respectively. Interestingly, one of the perturbing factors appears to be the steric hindrance of His307. Moreover, the protonation state of this residue is sensitive to slight variations around physiological pH and these changes may enhance local strain within the coiled‐coil, destabilizing inter‐subunit packing. This localized perturbation suggests a structural basis for the preferential heterotrimeric assembly observed in the presence of TRAF2 and TRAF1: (TRAF2)2/TRAF1 (Zheng et al. 2010). However, our PROPKA3 analysis does not predict a substantial change in its protonation state between pH 7.0 and pH 5.7. We therefore do not attribute the experimentally observed low‐pH destabilization specifically to protonation of His307, although this region may represent a structurally sensitive element of the trimeric assembly. In this context, a partially relaxed or asymmetric trimer may represent a more permissive state for subunit exchange, facilitating the destabilization of TRAF2 trimer and the incorporation of TRAF1 into the complex.

These structural effects are also reflected at the functional level. Indeed, membrane binding experiments indicate that TRAF2‐NC interacts more efficiently with GM1‐containing vesicles at acidic pH. Thanks to fluorescence anisotropy measurements, we provided evidence that the local protein dynamics increase at lower pH, upon dilution (Figure 6c). Since the aromatic residues (Trps and Tyrs) are all located in the protein C‐terminal region, this result suggests that the globular “heads” are less compact in an acid environment. Although moderate, the effect is reproducible and provides a possible explanation for the enhanced membrane binding capability. In fact, an increased flexibility and partial opening of the trimer would expose membrane‐interacting surfaces that are otherwise less accessible in a compact assembly, thus favoring interaction with the lipid bilayer. Since TNF signaling is strongly influenced by cellular stress conditions, these pH‐dependent conformational changes may contribute to the modulation of TRAF2 functional interactions during stress responses (Pasparakis and Vandenabeele 2015; Wajant et al. 2003). Although increased membrane association could, in principle, also arise from partial protein monomerization, this scenario appears unlikely for TRAF2‐NC. Indeed, as previously reported for a shorter TRAF2‐C construct (De Luca et al. 2024), monomerization is expected to cause a twofold reduction in the slow rotational component, Φ_2_, while we observed only a ~10% reduction at pH 5.7 and low protein concentration (Figure 6d). Thus, in the case of TRAF2‐NC, the presence of the TRAF2‐N domain appears to stabilize the trimer, even under conditions where some destabilization occurs. This suggests that the coiled‐coil region plays an important role not only in trimer formation, but also in modulating its stability and dynamics. Overall, these findings support a model in which TRAF2 acts as a dynamic scaffold capable of adapting its conformational ensemble in response to environmental changes, such as pH variations. This plasticity may be important to regulate the interactions with binding partners, particularly membrane receptors. As recently demonstrated by Lim and coworkers (Lim et al., 2025), rather than relying on complete dissociation, TRAF2 appears to modulate its functional state through subtle conformational rearrangements within the trimeric assembly. In this context, the ability of TRAFs to participate in both trimeric receptor complexes and pairwise interactions through their N‐terminal RING domains (Das et al. 2023) suggests a structurally adaptable architecture, which may be enabled by the peculiar, elongated shape of TRAFs.

In conclusion, TRAF2‐NC forms a stable yet adaptable trimer whose conformational plasticity likely contributes to the regulation of its interactions and cellular function.

## 4 MATERIALS AND METHODS

### 4.1 Expression and purification of TRAF2‐NC

The sequence coding for the residues 266–501 (TRAF2‐NC) of TRAF2 (Uniprot Q12933) was cloned in a pET21(a) vector. The sequence includes an N‐terminal methionine (residue 265) and a C‐terminal His‐tag (residues 502–516: LVPRGSLEHHHHHH; Mw 28.6 kDa). Plasmids were used to transform *Escherichia coli* strain C41(DE3) competent cells. Protein expression was induced by adding isopropyl‐beta‐D‐thiogalactopyranoside (IPTG) to a final concentration of 0.5 mM. Bacteria were grown in LB medium with chloramphenicol (34 μg mL^−1^) and ampicillin (100 μg mL^−1^). Cells were harvested and resuspended in a buffer containing 50 mM Tris–HCl pH 8.0, 500 mM NaCl, 120 μg mL^−1^ AEBSF, and protease inhibitors (cOmplete™ Protease Inhibitor Cocktail), treated with 4 μg mL^−1^ DNase, 40 mM MgSO_4_ and 50 μg mL^−1^ lysozyme, and disrupted by French press (30 KPsi) and sonication (20–30% amplitude, 45/15 s ON/OFF). After elimination of debris by high‐speed centrifugation, TRAF2‐NC was purified using Ni‐NTA (HisTrap FF crude Cytiva®; 300 mM imidazole for elution), followed by size exclusion chromatography (Superdex 200 16/600 pg, Cytiva®; 50 mM BIS‐TRIS propane pH 7.4, 200 mM NaCl, 10 mM dithiothreitol (DTT)). The concentration in terms of monomeric units of TRAF2‐NC was calculated by measuring the absorbance at 280 nm and considering a molar extinction coefficient of 17,780 M^−1^ cm^−1^.

### 4.2 Thermal stability assays

Thermal denaturation assays were conducted in a StepOne Real Time PCR System (Applied biosystem Thermo Fisher Scientific), with protein concentration of 0.85 mg mL^−1^ in different buffer conditions (0–500 mM NaCl, pH from 4.0 to 9.0). Samples were heated from 20 to 95°C (0.2°C min^−1^), and fluorescence intensity was measured using the Sypro Orange excitation/emission ranges of 470–505/540–700 nm, respectively. Experiments were performed both before and after freezing for storage at −80°C.

### 4.3 Liposome preparation

Aqueous dispersions of liposomes (LUVs) were prepared as follows: a film of lipids was prepared on the inside wall of a round bottom flask by evaporation of a 1‐mL chloroform solution containing the proper amounts of lipids (POPC) and pyrene‐GM1 1% from Sigma‐Aldrich. The films obtained were stored in a desiccator overnight under reduced pressure, then 1 mL of buffer solution (200 mM NaCl and 50 mM Tris–HCl pH 7.4 or phosphate pH 5.7) was added to obtain a lipid dispersion. The solution was vortex‐mixed and then freeze‐thawed six times from liquid nitrogen to 30°C. Dispersion was then extruded (10 times) through a 100‐nm polycarbonate membrane in order to obtain large unilamellar vesicles (LUVs). Extrusions were carried out at 30°C. The vesicle size was determined by dynamic light scattering measurements using a Horiba LB550 nanoparticle size analyzer.

### 4.4 Flow‐induced dispersion analysis

Flow‐induced dispersion analysis (FIDA) was performed on a Fida‐1 instrument (FidaBio) with a 75 μm × 1 m capillary. The capillary was washed with 1M NaOH and then with distilled water. The protein was dissolved in Tris–HCl 50 mM, 200 mM NaCl buffer at pH 7.4. In each experiment, the sample volume was 40 nL and the pressure injection 400 mbar. The protein elution profile was obtained by measuring the intrinsic fluorescence (excitation 280 nm) and the corresponding hydrodynamic radius was evaluated by the accompanying FIDA analysis software (FidaBio).

### 4.5 Analytical size exclusion chromatography

Experiments were performed at a protein concentration ~10 μM in a Superdex 200 increase 10/300 gL (Cytiva®) at 8°C with a flow rate of 0.8 mL min^−1^, using the following buffers:

1. 50 mM BIS‐TRIS propane pH 7.4, 200 mM NaCl, 10 mM DTT.
2. 45 mM KH_2_PO_4_ pH 5.7, 500 mM NaCl, 10 mM DTT.

Calibration was carried out with the protein standards: thyroglobulin (Mw = 669 kDa), conalbumin (Mw = 75 kDa), and ribonuclease A (Mw = 13 kDa) (Figure S2).

### 4.6 Dynamic light scattering

Purified proteins were centrifuged at 13,000*g* for 10 min at 4°C prior to dynamic light scattering (DLS) analysis. All measurements were performed in triplicates at 10 and 20°C in a PUNK DLS Protein Sizing System‐Unchained Labs® with a data acquisition time of 30 s for each measure, for 20 measurements.

Light scattering measurements were performed on a Horiba (Kyoto, Japan) LB‐500 nanoparticle size analyzer, equipped with a 650‐nm, 5 mW laser diode. The analysis of the raw data was carried out using the accompanying software, which is based on a Fourier‐transform deconvolution procedure. Measurements were performed in triplicate at 20°C and at different protein concentrations as specified in the text. The heterogeneity of the diameter (*D*_*i*_) distribution was assessed evaluating the particle dispersity using (by analogy with the mass dispersity function in polymer science) the expression,

$$\text{Particle dispersity}=\frac{\sum_{i}{\left(D_{i}-<D>\right)}^{2}q\left(i\right)}{100<D{>}^{2}},$$

where $<D>=\sum_{i}D_{i}\;q\left(i\right)/100$, the *q*(*i*) being the *i*th DLS experimental population, characterized by a diameter *D*_*i*_.

### 4.7 Circular dichroism

Circular dichroism (CD) spectra as a function of the temperature were recorded on a J‐1100ST spectropolarimeter (Jasco Inc., Japan) equipped with a Peltier temperature control system. Measurements were carried out in quartz cuvettes with a 0.1‐cm path length, using a protein concentration of 2.0 μM in 50 mM BIS‐TRIS propane pH 7.4, 200 mM NaCl, 10 mM DTT and using a protein concentration of 1.2 μM in KH_2_PO_4_ pH 5.7, 150 mM NaCl, 5 mM DTT.

Far‐UV spectra (180–260 nm) were collected at a scanning speed of 20 nm min^−1^, response time of 1 s, and a bandwidth of 1 nm. Each spectrum was averaged over three scans to reduce noise. Buffer spectra were subtracted, and the results were expressed as mean residue molar ellipticity (*θ*, deg cm^2^ dmol^−1^).

For thermal stability assays, the signal at 218 nm was monitored for TRAF2‐NC at pH 7.4 and 222 nm for TRAF2 at pH 5.7 as a function of temperature (20–95°C, with a heating rate of 1°C min^−1^).

Circular dichroism spectra of TRAF2‐NC at different concentrations and pH (7.4 or 5.7) were performed on a Jasco J‐815 spectropolarimeter at 20°C, using a 1‐mm pathlength quartz cuvette. All the data were corrected for buffer contributions and converted to molar ellipticity.

Secondary structure contents were estimated using the BeStSel server (Micsonai et al. 2018) and the relative α‐helix and β‐sheet content was quantified at each dilution point.

### 4.8 Fluorescence spectroscopy

Steady‐state fluorescence measurements were performed with the ISS PC1 spectrofluorometer (ISS, Inc., Champaign, IL) maintaining the samples at 20°C by an external bath circulator. The binding of TRAF2‐NC to LUVs was monitored through the energy transfer between tryptophans and pyrene, varying the [lipid]/[TRAF2‐NC] ratio. The Δ*F* = *F*_0_ − *F*_*r*_ difference between the area at [LUVs] = 0, *F*_0_, and those collected at increasing lipid concentration, *F*_*r*_, was plotted and interpolated with a hyperbolic function, obtaining the asymptotic value, Δ*F*_max_. The relative fluorescence change Δ*F*/Δ*F*_max_ was fitted using a variant of the theoretical approach developed by Tatulian and co‐workers (Qin et al. 2004) and used in the past for other membrane‐binding proteins (Muroni et al. 2024; Ripanti et al., 2021). In particular,

$$\frac{∆F}{∆F_{\max}}=b-\sqrt{b^{2}-\frac{\delta \left[L_{\mathrm{tot}}\right]}{N\;\left[P\right]}},$$

with

$$b=\frac{1}{2}\left(1+\frac{\delta \left[L_{\mathrm{tot}}\right]}{N\left[P\right]}+\frac{K_{D}}{\left[P\right]}\right),$$

$$K_{D}=\frac{\delta }{N}L_{\frac{1}{2}}-\frac{P}{2},$$

where *N* is the average number of lipid “hidden” by TRAF2‐NC association with the bilayer, *K*_D_ is the apparent dissociation constant, $L_{\frac{1}{2}}$ is the lipid concentration corresponding to half‐saturation of binding and *δ*[*L*_tot_] is the fraction of total lipids observable upon binding, that is, those of the external leaflet of the LUVs and, among them, the fraction within a distance of about 20 Å far apart from the pyrene molecule.

Fluorescence lifetimes and dynamic anisotropy measurements were carried out using the phase shift and demodulation technique (Lakowicz et al. 1984) with a KOALA‐ISS fluorimeter (ISS, Champaign, IL). Excitation was provided by a 280‐nm laser diode, while emission was collected through a 305‐nm long‐pass filter to minimize scattering contributions.

Anisotropy decays were recorded using Glan–Thompson polarizers, applying the appropriate G‐factor correction. For each sample, three independent acquisitions of phase shift and demodulation data were collected over 20 modulation frequencies. Data fitting was performed with Vinci software (ISS Champaign, IL), by comparing experimental data with anisotropy decay models using a Levenberg–Marquardt algorithm for *χ* ^2^ minimization. In this case, the best fit was obtained with a two‐component rotational decay model.

### 4.9 Small angle X‐ray scattering

All experiments were performed at the European Synchrotron Radiation Facility (ESRF) in Grenoble at the BM29 (MX‐2591 and MX‐2790) beamline with X‐ray energy 12.5 keV and detector Pilatus3 2M in batch or SEC‐SAXS mode, using a Superdex 200 increase 10/300 column (Cytiva(R)) in buffer 50 mM BIS‐TRIS propane pH 7.4, 200 mM NaCl, 10 mM DTT or AdvanceBio SEC 130 Å column (4.6 × 300 mm; Agilent Technologies), in buffer 200 mM NaCl and 50 mM phosphate pH 5.7. SEC‐SAXS data were analyzed with the ATSAS software package (Manalastas‐Cantos et al. 2021) with programs *chromix* (Panjkovich and Svergun 2018) and *primus* and the low‐resolution models were generated with the program *Dammin* (Svergun 1999); high‐resolution model of the protein was obtained by AlphaFold3 (Abramson et al. 2024) and its analysis including normal mode structural variation with *sreflex* software (Panjkovich and Svergun 2016).

### 4.10 SAXS‐restrained and unbiased‐MD model refinement

A trimeric model of the human TNF receptor‐associated factor 2 (TRAF2), residues 266–501 (UniProt accession code Q12933), was generated using AlphaFold3 (Abramson et al. 2024). The input sequence, submitted in three copies, is the same as the expressed proteins including the N‐terminal methionine (residue 265) and the C‐terminal His‐tag. The five models generated were very similar (RMSD <1 Å) and had the same template modeling (pTM = 0.85) and interface predicted template modeling (ipTM = 0.84) scores. The models differed mainly for the conformation of the C‐terminal His‐tag: in four of the models, the GSLEHHHHHH tag in each protomer was predicted to be an α‐helix, while in one it appeared as a flexible, disordered extension; the latter model was chosen as it is more realistic for an affinity tag and facilitates SAXS‐restrained refinement by avoiding the need to disrupt a pre‐formed helix. Figure S3 provides the AF3 prediction confidence (pLDDT) along the sequence. The whole TRAF trimer is predicted with high confidence, with the exception of the His‐tag region.

The protonation states of the titratable residues were assigned at pH 7.4 ± 0.5 using the Protein Preparation Wizard (Schrödinger Maestro Suite, version 14.2.118, release 2024–4) (Madhavi Sastry et al. 2013), as reported in Table S2. The PROPKA3 method (Olsson et al. 2011) predicts barely any protonation changes between pH 7 and pH 5.7 beyond the His‐tag (Figure S4). The resulting structure was used to build the solvated system with the Solution Builder tool from the CHARMM‐GUI webserver (Lee et al. 2016). The system was solvated with TIP3P water and 200 mM NaCl in a 200 (*x*) × 120 (*y*) × 120 (*z*) Å^3^ periodic box.

Energy minimization was performed using the steepest descent algorithm in GROMACS 2024.5 (Van Der Spoel et al., 2005), with the CHARMM36m force field (Huang et al. 2017). Positional restraints were applied to backbone and side‐chain atoms with force constants of 400 and 40 kJ mol^−1^ nm^−2^, respectively. Electrostatics were treated using the Particle Mesh Ewald (PME) method with a 1.2‐nm cut‐off, and van der Waals interactions were modeled using a force‐switch scheme between 1.0 and 1.2 nm. The system was considered minimized when the maximum force fell below 100 kJ mol^−1^ nm^−1^. All bonds involving hydrogen atoms were constrained using the LINCS algorithm.

Equilibration was performed under NPT conditions for 100 ns. A 2‐fs time step was used, and positional restraints were maintained. The temperature was kept at 300 K using the velocity‐rescaling thermostat with a coupling constant of 0.2 ps. Pressure was maintained at 1 bar using the isotropic C‐rescale barostat with a time constant of 5 ps and a compressibility of 4.5 × 10^−5^ bar^−1^. Electrostatics, PME, van der Waals, and hydrogen‐containing bonds were modeled consistently with the minimization procedure.

A 7‐ns refinement simulation was subsequently performed with SAXS restraints using GROMACS 2024.5 patched with PLUMED 2.11 (Tribello et al. 2025). This restrained (biased) simulation was intended for SAXS‐guided structural refinement rather than equilibrium conformational sampling; therefore, independent replicas were not performed for this refinement stage, and the simulation length is not related to actual relaxation timescales. The experimental SAXS data were pre‐processed using ATSAS (Manalastas‐Cantos et al. 2021), and 35 intensity values were extracted over the ~0 to ~0.3 Å^−1^ momentum transfer (*q*) range. The hybrid‐resolution one‐bead hySAS method (Ballabio et al. 2023) was used to compute SAXS intensities on‐the‐fly during the simulation. A linear restraint was applied on the correlation between experimental and simulated intensity profiles. To avoid translation and rotation of the protein within the box, additional restraints were applied to the *z* and *y* components of the geometric centers defined by the Cα atoms of residues S274‐E283 in chains A, B, and C (center 1) and of residues K331‐Q340 in chains A, B, and C (center 2). The atomic coordinates of the protein were saved only when the real‐time correlation exceeded 0.999901. All saved conformations were re‐analyzed with PEPSI‐SAXS (Grudinin et al. 2017), accounting for explicit hydrogen atoms, to validate agreement with the experimental dataset (*q* range ~0 to ~0.5 Å^−1^).

Because the SAXS‐restrained simulation was used as a data‐guided structural refinement rather than for equilibrium conformational sampling, conventional MD convergence criteria were not applied. Refinement quality was instead assessed from agreement with the experimental SAXS profile. The representative model was selected as the retained conformation yielding the best agreement with the experimental SAXS curve upon PEPSI‐SAXS analysis, rather than as the final frame of the trajectory. The simulation conditions, including force field parameters and settings, were consistent with the equilibration step.

Three independent unbiased (i.e., not informed by SAXS measurements) MD simulations were performed as controls, each for 300 ns. The replicas were initiated using different randomly assigned initial velocities. The system was prepared analogously as before, but without any biasing force or orientational restraint, now within a larger 195 × 195 × 195 Å^3^ box (containing 698,136 atoms including solvent).

## AUTHOR CONTRIBUTIONS

**Federica Cossu:** Funding acquisition; investigation; writing – review and editing; data curation; methodology; formal analysis; project administration; conceptualization; supervision; resources. **Serena Virili:** Methodology; validation; formal analysis; data curation; investigation; writing – review and editing. **Bianca Braggiotti:** Investigation; methodology; validation; formal analysis; data curation. **Francesco Bonì:** Methodology; validation; visualization; formal analysis; data curation. **Federico Ballabio:** Methodology; formal analysis; data curation; software; validation. **Toni Giorgino:** Writing – review and editing; funding acquisition; formal analysis; data curation; validation; supervision. **Mario Milani:** Conceptualization; investigation; writing – review and editing; methodology; validation; formal analysis; data curation. **Giampiero Mei:** Writing – original draft; methodology; project administration; conceptualization; funding acquisition; supervision; resources; writing – review and editing. **Almerinda Di Venere:** Data curation; funding acquisition; writing – original draft; conceptualization; validation; formal analysis; investigation; supervision; resources; project administration. **Eloise Mastrangelo:** Conceptualization; investigation; funding acquisition; writing – original draft; validation; formal analysis; supervision; data curation; project administration; resources; writing – review and editing.

## Supporting information


**Figure S1.** SDS‐PAGE of TRAF2‐NC. Lane 1: marker; Lane 2: TRAF2‐NC after Ni‐NTA; Lane 4: TRAF2‐NC after Superdex 200.
**Figure S2.** Superdex 200 Calibration Curves. Calibration curves using in a Superdex 200 increase 10/300 gL (Cytiva®) at 8°C with a flow rate of 0.8 mL min^−1^: (a) at pH = 5.7 in the buffer 45 mM KH2PO4 pH 5.7, 500 mM NaCl, 10 mM DTT; (b) at pH = 7.4 in the buffer 50 mM BIS‐TRIS propane pH 7.4, 200 mM NaCl, 10 mM DTT.
**Figure S3.** Per‐residue confidence profile of the AlphaFold‐derived TRAF2‐NC model. Per‐residue confidence profile of the AlphaFold3‐derived TRAF2‐NC model. Residue number is plotted on the x‐axis and the AlphaFold predicted Local Distance Difference Test (pLDDT) score on the y‐axis. Higher pLDDT values indicate greater confidence in the predicted local structure.
**Figure S4.** Per‐residue predicted pKa values. No significant protonation changes are expected between pH 7 and pH 5.7 beyond the HIS‐tag region.
**Figure S5.** Chi‐squared values of individual trajectory frames along the three unbiased simulation replicas with respect to experimental SAXS data (computed by Pepsi‐SAXS). Minima are highlighted.
**Table S1.** Screening of buffer conditions by thermal stability assays.
**Table S2.** Assigned protonation states of histidine residues in the TRAF2‐NC model.

## Data Availability

The initial AlphaFold‐derived TRAF2‐NC model, the SAXS‐refined structural model, and the three unbiased simulation trajectories of 300 ns each have been deposited in Zenodo at https://zenodo.org/, https://doi.org/10.5281/zenodo.22112875. ESRF data are available as: mx‐2591, https://doi.org/10.15151/ESRF‐ES‐1471046304; mx‐2790, https://doi.org/10.15151/ESRF-ES-2390592976.
