# Supplementary material for: Structural dynamics and pH‐regulated membrane binding of the TRAF2‐NC trimer
Source: Protein Sci. 2026 Oct 6;35(11):e70817. doi: 10.1002/pro.70817 (PMC13641095; doi:10.1002/pro.70817)
Supplement: Supplementary file 1 [file PRO-35-e70817-s001.pdf]

Structural dynamics and pH-regulated membrane binding of the TRAF2-NC trimer

Federica Cossu[1,*], Serena Virili[2,*], Bianca Braggiotti[1], Francesco Bonì[1], Federico Ballabio[1,3], Toni Giorgino[1], Mario Milani[1], Giampiero Mei[2], Almerinda Di Venere[+,2], Eloise Mastrangelo[+,1]

[1]Institute of Biophysics, National Research Council (IBF-CNR), Via Corti 12, 20133 Milan, Italy

[2]Department of Experimental Medicine, Tor Vergata University of Rome, Via Montpellier 1, 00133 Rome, Italy

[3]Institute of Structural Biology, Molecular Targets and Therapeutics Center, Helmholtz Munich - Deutsches Forschungszentrum Für Gesundheit Und Umwelt (GmbH), D-85764, Neuherberg, Germany

The supplementary materials concern:

Figure S1. SDS-PAGE of TRAF2-NC.

Figure S2. Superdex 200 Calibration Curves.

Figure S3. Per-residue confidence profile of the AlphaFold-derived TRAF2-NC model

Figure S4. Per-residue predicted pKa values.

Figure S5. Chi-squared values of individual trajectory frames along the three unbiased simulation replicas with respect to experimental SAXS data (computed by Pepsi-SAXS).

Table S1. Screening of buffer conditions by thermal stability assays.

Table S2. Assigned protonation states of histidine residues in the TRAF2-NC model.

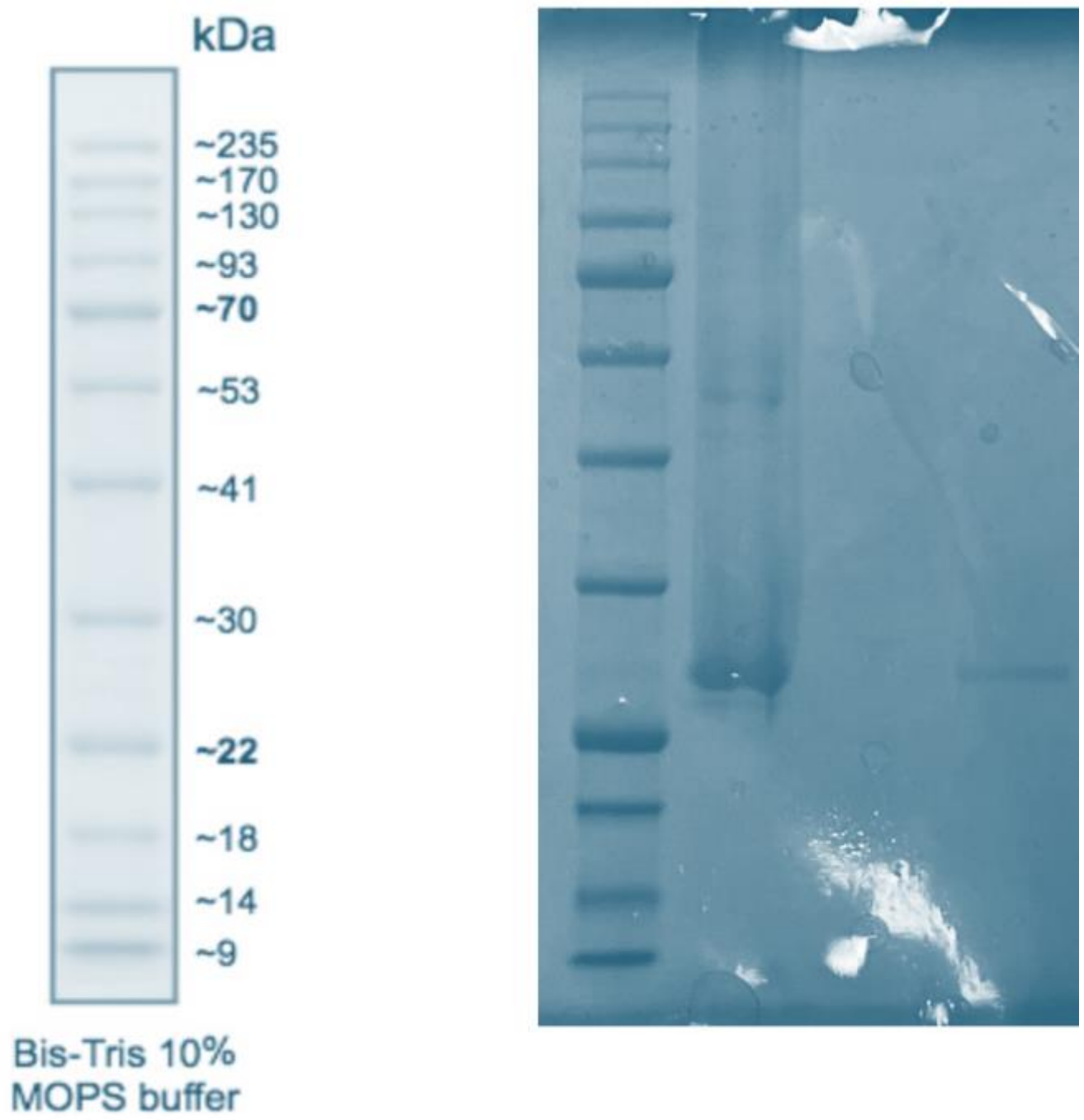

Figure S1. SDS-PAGE of TRAF2-NC. Lane 1: marker; Lane 2: TRAF2-NC after Ni-NTA; Lane 4: TRAF2-NC after Superdex 200.

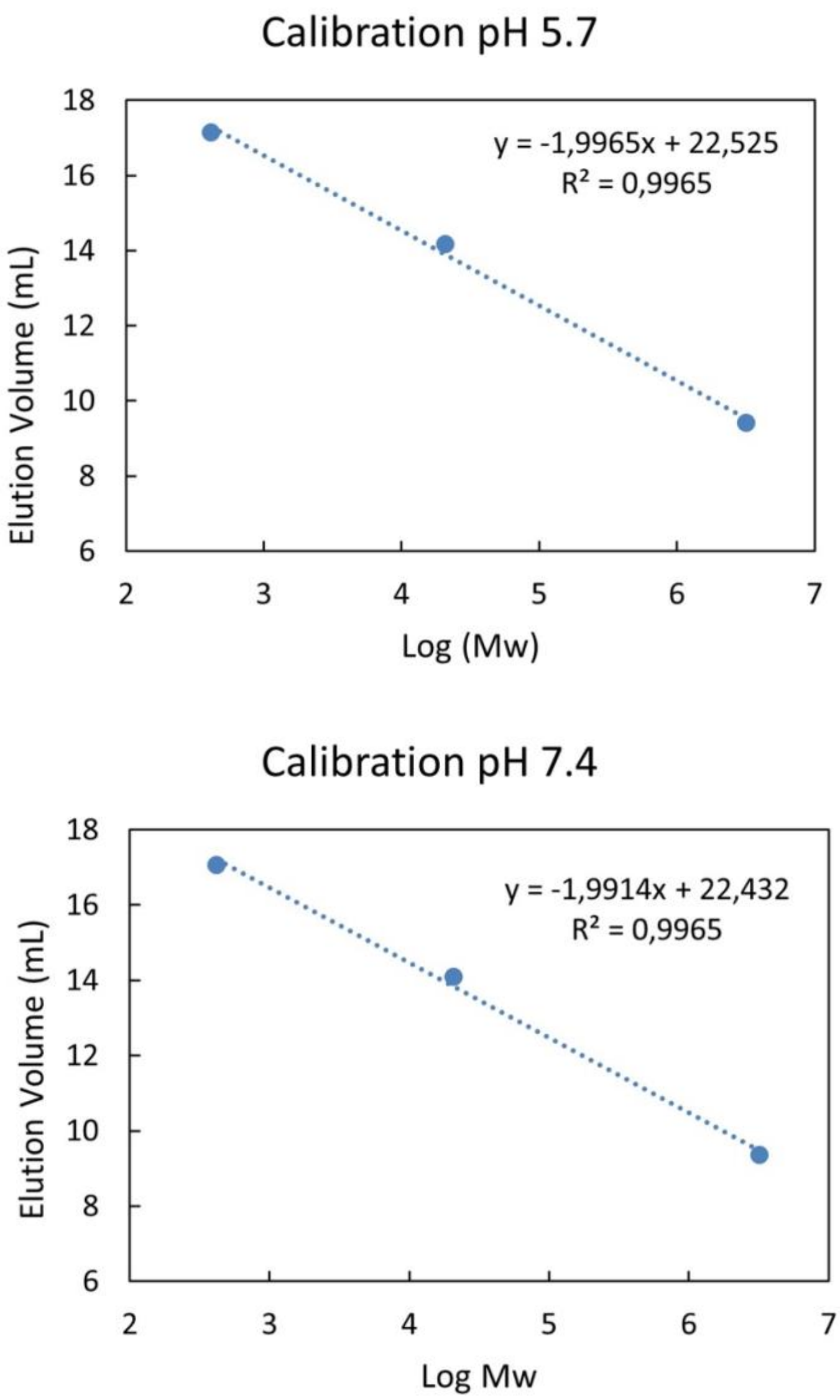

Figure S2. Superdex 200 Calibration Curves. Calibration curves using in a Superdex 200 increase 10/300 gl (Cytiva ®) at 8 °C with a flow rate of 0.8 ml/min: A) at pH=5.7 in the buffer 45 mM KH2PO4 pH 5.7, 500 mM NaCl, 10 mM DTT; B) at pH=7.4 in the buffer 50 mM BIS-TRIS propane pH 7.4, 200 mM NaCl, 10 mM DTT.

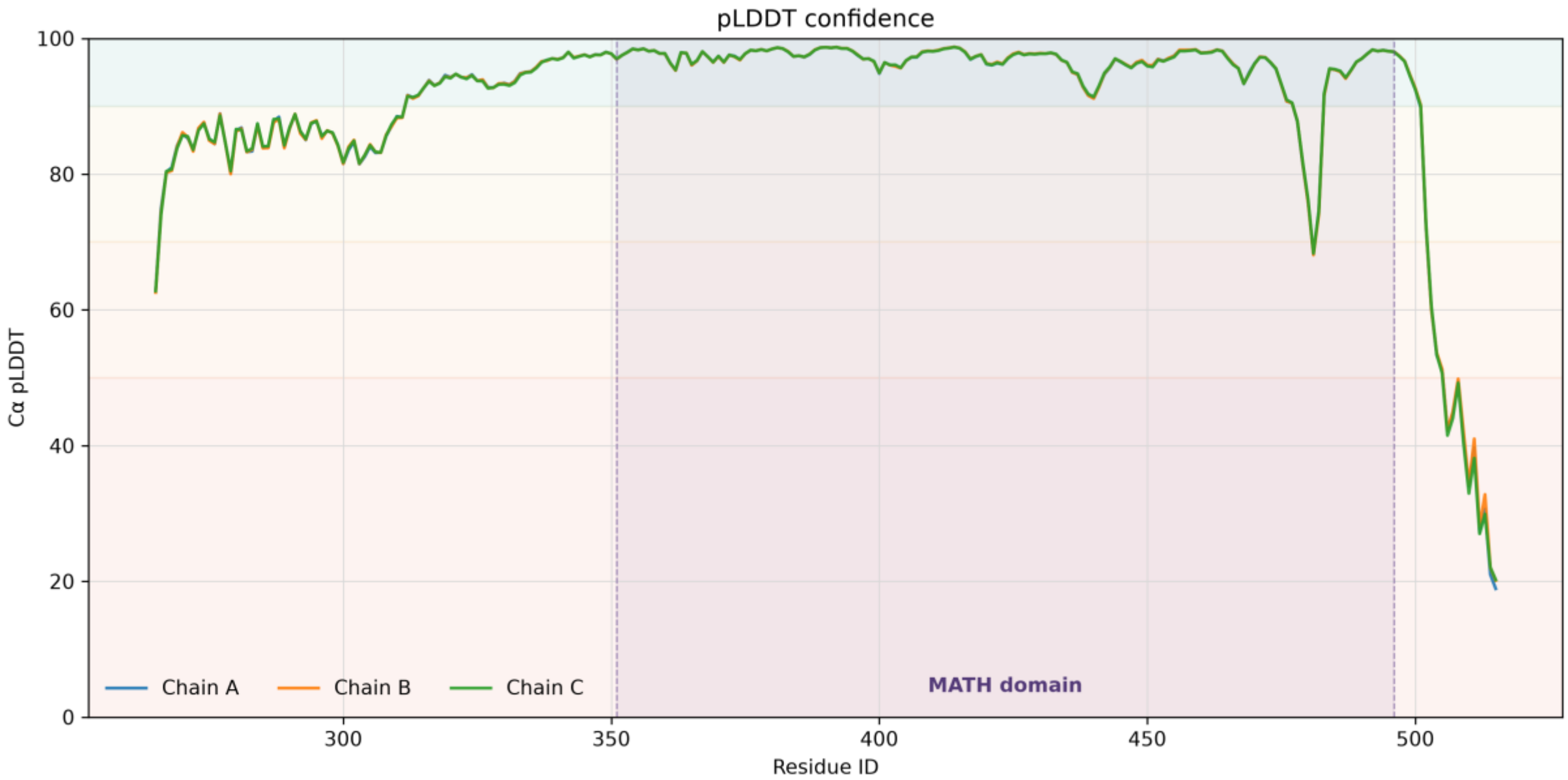

Figure S3. Per-residue confidence profile of the AlphaFold-derived TRAF2-NC model. Per-residue confidence profile of the AlphaFold3-derived TRAF2-NC model. Residue number is plotted on the *x*-axis and the AlphaFold predicted Local Distance Difference Test (pLDDT) score on the *y*-axis. Higher pLDDT values indicate greater confidence in the predicted local structure.

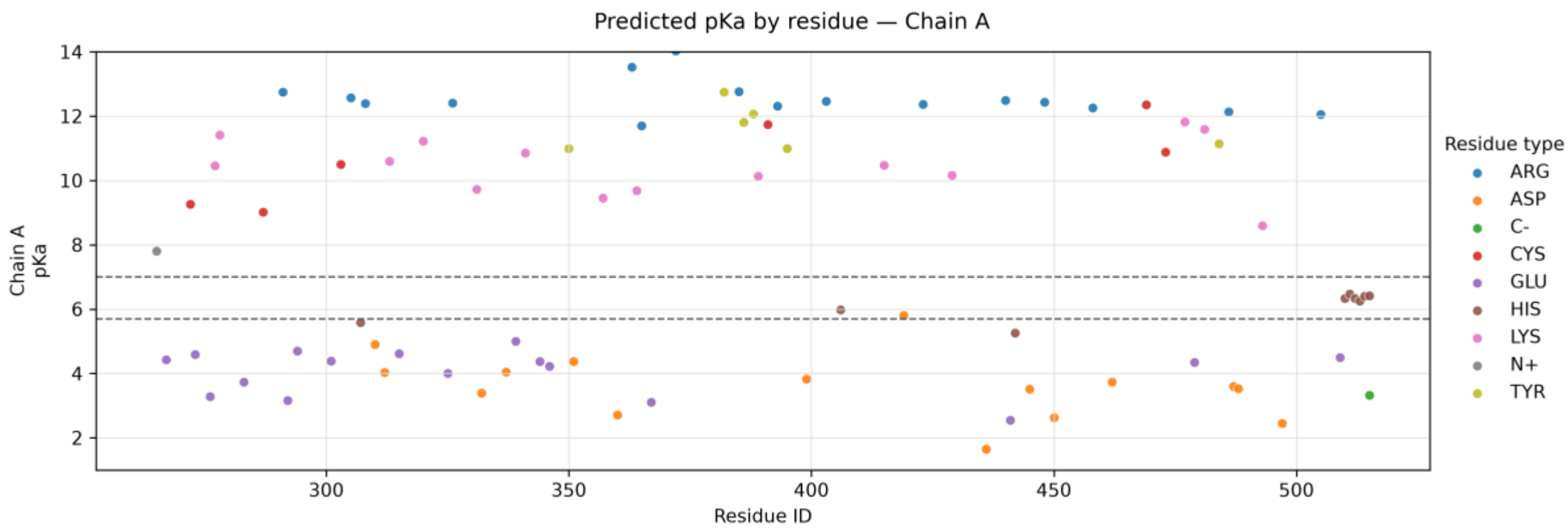

Figure S4. Per-residue predicted pKa values. No significant protonation changes are expceded between pH 7 and pH 5.7 beyond the HIS-tag region.

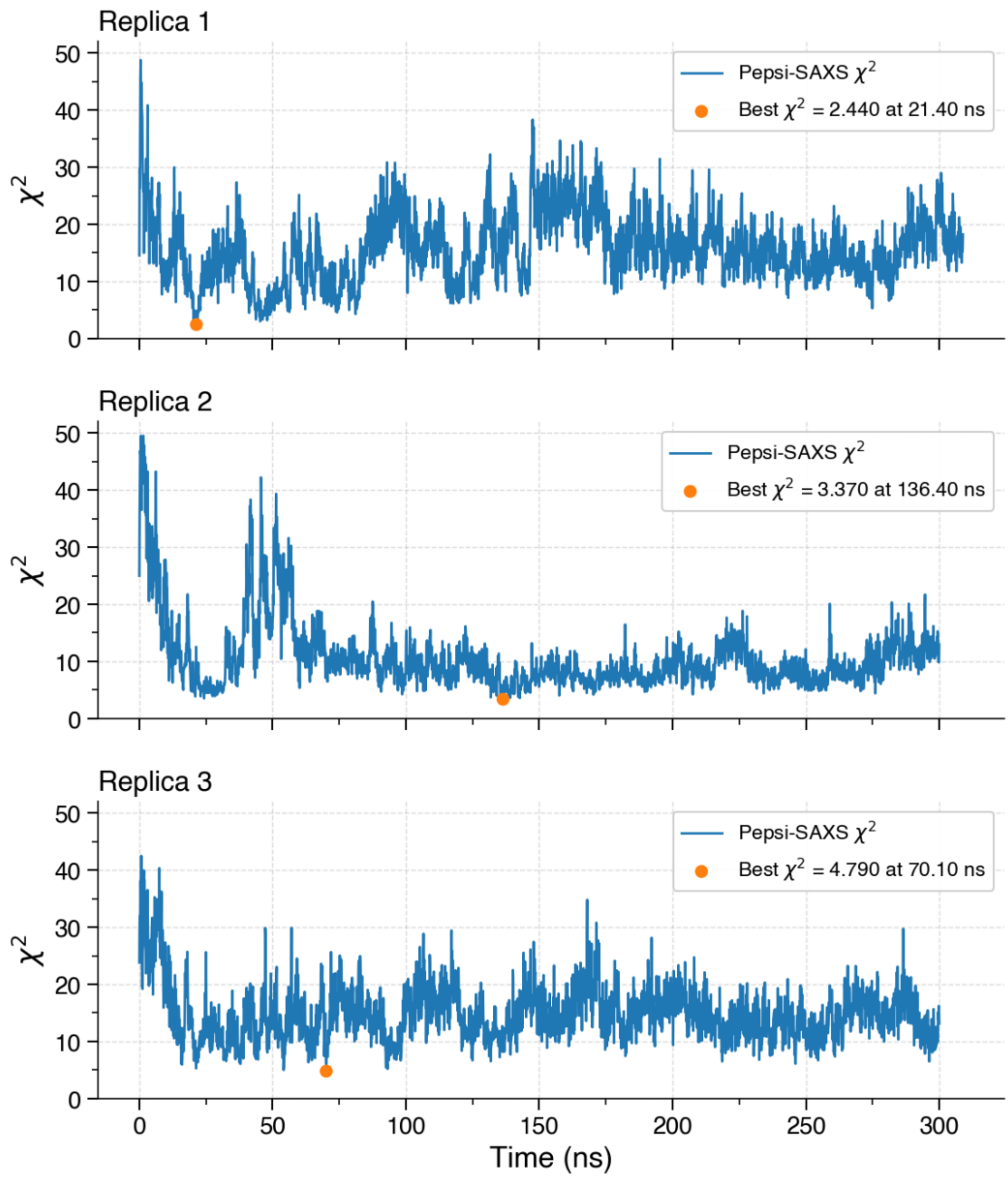

Figure S5. Chi-squared values of individual trajectory frames along the three unbiased simulation replicas with respect to experimental SAXS data (computed by Pepsi-SAXS). Minima are highlighted.

Table S1. Screening of buffer conditions by thermal stability assays.

0.1 M	*pH*	*NaCl [M]**				
		0	*0.1*	*0.2*	*0.3*	*0.5*
Sodium Citrate	*4.0*	46.3	45.2	44.3	43.5	41.8
Potassium Phosphate	*5.7*	53.7	53.1	52.6	52.1	51.5
MES	*6.0*	52.1	51.9	51.5	51.7	51.0
Bis Tris Propane	*7.4*	55.3	54.9	54.5	54.4	53.8
Tris-HCl	*8.0*	55.7	55.1	54.8	54.4	54.0
Tris-HCl	*9.0*	54.6	54.1	53.7	53.3	52.8

*The experimental error is based on three different measurements and is approximately ±0.1–0.3.

Table S2. Assigned protonation states of histidine residues in the TRAF2-NC model.

Residue	Chain	PROPKA3-predicted pKa	Assigned state	Protonated ring nitrogen
His307	A	5.59	HID	Nδ1
His406	A	5.98	HID	Nδ1
His442	A	5.26	HIE	Nε2
His510*	A	6.34	HID	Nδ1
His511*	A	6.47	HID	Nδ1
His512*	A	6.33	HID	Nδ1
His513*	A	6.26	HID	Nδ1
His514*	A	6.40	HID	Nδ1
His515*	A	6.42	HID	Nδ1
His307	B	6.06	HID	Nδ1
His406	B	5.98	HID	Nδ1
His442	B	5.23	HIE	Nε2
His510*	B	6.36	HID	Nδ1
His511*	B	6.46	HID	Nδ1
His512*	B	6.32	HID	Nδ1
His513*	B	6.25	HID	Nδ1
His514*	B	6.40	HID	Nδ1
His515*	B	6.41	HID	Nδ1
His307	C	5.36	HID	Nδ1
His406	C	5.98	HID	Nδ1
His442	C	5.23	HIE	Nε2
His510*	C	6.34	HID	Nδ1
His511*	C	6.46	HID	Nδ1
His512*	C	6.34	HID	Nδ1
His513*	C	6.26	HID	Nδ1
His514*	C	6.40	HID	Nδ1
His515*	C	6.41	HID	Nδ1

HID and HIE denote neutral histidine tautomers protonated at Nδ1 and Nε2, respectively; HIP denotes the positively charged form protonated at both ring nitrogens. No HIP residues were assigned in this model. * indicates residues in the His-tag.
